# Supplementary material for: Prenatal fortified balanced energy-protein supplementation and birth outcomes in rural Burkina Faso: A randomized controlled efficacy trial
Source: PLoS Med. 2022 May 13;19(5):e1004002. doi: 10.1371/journal.pmed.1004002 (PMC9140265; doi:10.1371/journal.pmed.1004002)
Supplement: S2 Table — (DOCX) [file pmed.1004002.s003.docx]

**Prenatal fortified balanced energy-protein supplementation and birth outcomes in rural Burkina Faso: A randomised controlled efficacy trial**

**Supplementary table**

**S2 Table. Per protocol analyses of primary and secondary outcomes**

| **Birth characteristics** | **Control**^a^  **(*n* = 850)** | **Intervention**^a^**^,b^**  **(*n* = 631)** | **Unadjusted ∆**^c^  **(95% CI)** | **p value** | **Adjusted ∆**^c^  **(95% CI)** | **p value** |
| --- | --- | --- | --- | --- | --- | --- |
| Small-for-gestational age | 236 (27.8) | 155 (24.6) | -3.30 (-7.89, 1.29) | 0.16 | -3.16 (-7.59, 1.26) | 0.161 |
| Large-for-gestational age | 13 (1.53) | 10 (1.58) | 0.07 (-1.20, 1.35) | 0.90 | 0.08 (-1.19, 1.35) | 0.90 |
| Low birth weight | 103 (12.1) | 52 (8.24) | -3.66 (-6.69, -0.63) | 0.018 | -3.85 (-6.78, -0.92) | 0.010 |
| Preterm delivery | 39 (4.59) | 21 (3.33) | -1.31 (-3.33, 0.71) | 0.20 | -1.47 (-3.49, 0.56) | 0.16 |
| Gestational age, weeks | 39.9 ± 1.78 | 40.0 ± 1.54 | 0.20 (0.02, 0.38) | 0.033 | 0.20 (0.02, 0.38) | 0.027 |
| Birth weight, g | 2988 ± 450 | 3037 ± 430 | 45.3 (-0.50, 91.0) | 0.05 | 45.0 (2.52, 87.5) | 0.038 |
| Birth length, cm | 48.2 ± 2.25 | 48.3 ± 2.17 | 0.18 (-0.04, 0.40) | 0.11 | 0.18 (-0.02, 0.39) | 0.08 |
| Ponderal index^d^ | 26.6 ± 2.67 | 26.8 ± 2.65 | 0.14 (-0.11, 0.40) | 0.27 | 0.14 (-0.12, 0.39) | 0.29 |
| Head circumference, cm | 33.4 ± 1.64 | 33.5 ± 1.56 | 0.12 (-0.04, 0.28) | 0.14 | 0.13 (-0.03, 0.28) | 0.10 |
| Thoracic circumference, cm | 31.7 ± 1.84 | 32.0 ± 1.71 | 0.22 (0.04, 0.40) | 0.019 | 0.22 (0.05, 0.39) | 0.013 |
| Arm circumference, cm | 100 ± 8.42 | 101 ± 8.38 | 0.92 (0.10, 1.75) | 0.029 | 0.98 (0.20, 1.76) | 0.014 |

^a^Values are frequencies (%) or mean ± standard deviation.

^b^Subsample of women meeting a strict compliance rate ≥75%. Strict compliance was defined as the number of days with observed balanced energy-protein supplement intake over the total days between study inclusion and delivery.

^c^Unadjusted and adjusted group differences (∆) were estimated by fitting linear regression models for the continuous outcomes, to estimate the mean group difference, and using linear probability models with robust variance estimators for the binary outcomes, to estimate risk difference in percentage points. All models contained health centre and randomization block as fixed effect to account for clustering by the study design. Adjusted models additionally contained a priori set known prognostic factors of birth outcome including maternal age, primiparity, gestational age, height, mid-upper arm circumference, body mass index, and haemoglobin level at study enrolment.

^d^Ponderal index calculated as birth weight in g / (birth length in cm)^3^ × 1000.

CI, confidence interval.
