# Supplementary material for: Prenatal fortified balanced energy-protein supplementation and birth outcomes in rural Burkina Faso: A randomized controlled efficacy trial
Source: PLoS Med. 2022 May 13;19(5):e1004002. doi: 10.1371/journal.pmed.1004002 (PMC9140265; doi:10.1371/journal.pmed.1004002)
Supplement: S3 Table — SGA, small-for-gestational age. (DOCX) [file pmed.1004002.s004.docx]

**Prenatal fortified balanced energy-protein supplementation and birth outcomes in rural Burkina Faso: A randomised controlled efficacy trial**

**Supplementary table**

**S3 Table. Subgroup analysis by potential treatment effect modifiers of small-for-gestational age**

| **Subgroup factor** | **Control**^a^  **(*n* = 850)** | **Intervention**^a^  **(*n* = 809)** | **Unadjusted ∆**^b^  **(95% CI)** | **p value** | **Adjusted ∆**^b^  **(95% CI)** | **p value** |
| --- | --- | --- | --- | --- | --- | --- |
| Maternal BMI^c^ |  |  |  | 0.14 |  | 0.26 |
| <18.5 kg/m² (underweight) | 55 (6.48) | 57 (7.05) | - | - | - | - |
| ≥ 18.5 kg/m² | 795 (93.5) | 752 (93.0) | - | - | - | - |
| Maternal haemoglobin level^c^ |  |  |  | 0.050 |  | 0.036 |
| <11 g/dl (anaemic) | 309 (36.4) | 309 (38.2) | 2.06 (-5.41, 9.53) | 0.59 | 3.27 (-4.02, 10.6) | 0.38 |
| ≥11 g/dl | 541 (63.7) | 500 (61.8) | -7.14 (-12.4, -1.90) | 0.008 | -6.77 (-11.8, -1.71) | 0.009 |
| Maternal MUAC^c^ |  |  |  | 0.095 |  | 0.078 |
| <23 cm | 461 (54.2) | 460 (56.9) | 0.22 (-5.87, 6.32) | 0.94 | 1.16 (-4.70, 7.01) | 0.70 |
| ≥23 cm | 389 (45.8) | 349 (43.1) | -6.47 (-12.4, -0.50) | 0.034 | -6.07 (-11.9, -0.32) | 0.042 |
| Maternal height^c^ |  |  |  | 0.36 |  | 0.28 |
| <155 cm | 81 (9.53) | 80 (9.89) | - | - | - | - |
| ≥155 cm | 769 (90.5) | 729 (90.1) | - | - | - | - |
| Maternal age^c^ |  |  |  | 0.062 |  | 0.050 |
| <20 years | 185 (21.8) | 191 (23.6) | 2.70 (-8.05, 13.5) | 0.62 | 5.08 (-5.41, 15.6) | 0.34 |
| ≥20 years | 665 (78.2) | 618 (76.4) | -5.68 (-10.3, -1.07) | 0.016 | -5.23 (-9.74, -0.72) | 0.023 |
| Primiparity^c^ |  |  |  | 0.19 |  | 0.28 |
| Yes | 176 (20.7) | 181 (22.4) | - | - | - | - |
| No | 674 (79.3) | 628 (77.6) | - | - | - | - |
| Household food insecurity^c^ |  |  |  | 0.64 |  | 0.29 |
| Food insecure | 462 (54.4) | 445 (55.0) | - | - | - | - |
| Food secure | 388 (45.7) | 364 (45.0) | - | - | - | - |

| Depression possible^c^ |  |  |  | 0.25 |  | 0.35 |
| --- | --- | --- | --- | --- | --- | --- |
| Yes | 19 (2.2) | 14 (1.7) | - | - | - | - |
| No | 831 (97.8) | 795 (98.3) | - | - | - | - |
| Depression probable^c^ |  |  |  | 0.73 |  | 0.99 |
| Yes | 69 (8.1) | 60 (7.4) | - | - | - | - |
| No | 781 (91.9) | 749 (92.6) | - | - | - | - |
| Child sex^c^ |  |  |  | 0.16 |  | 0.08 |
| Female | 437 (51.4) | 50.3 | - | - | -6.73 (-12.6, -0.81) | 0.026 |
| Male | 413 (48.6) | 49.7 | - | - | 1.13 (-4.78, 7.04) | 0.71 |
| Season of delivery^c^ |  |  |  | 0.50 |  | 0.46 |
| Lean (June - September) | 267 (31.4) | 260 (32.1) | - |  | - |  |
| Plenty | 583 (68.6) | 549 (67.9) | - |  | - |  |
| Inter-pregnancy interval^c^ |  |  |  | 0.88 |  | 0.98 |
| <18 mo | 18 (2.12) | 27 (3.34) | - | - | - |  |
| ≥18 mo | 832 (97.9) | 782 (96.7) | - | - | - |  |

^a^Values are frequencies (%).

^b^Unadjusted and adjusted group differences (∆) were estimated by fitting linear probability models with robust variance estimators for the binary small-for-gestational age outcome, to estimate risk difference in percentage points. All models contained health centre and randomization block as fixed effect to account for clustering by the study design. Adjusted models additionally contained a priori set known prognostic factors of birth outcome including maternal age, primiparity, gestational age, height, mid-upper arm circumference, body mass index, and haemoglobin level at study enrolment

^c^Statistical significance was set at *P* < 0.10 for interaction.

BMI, body mass index; CI, confidence interval; MUAC, mid-upper arm circumference.
