# Supplementary figures and images for: Prenatal fortified balanced energy-protein supplementation and birth outcomes in rural Burkina Faso: A randomized controlled efficacy trial
Source: PLoS Med. 2022 May 13;19(5):e1004002. doi: 10.1371/journal.pmed.1004002 (PMC9140265; doi:10.1371/journal.pmed.1004002)

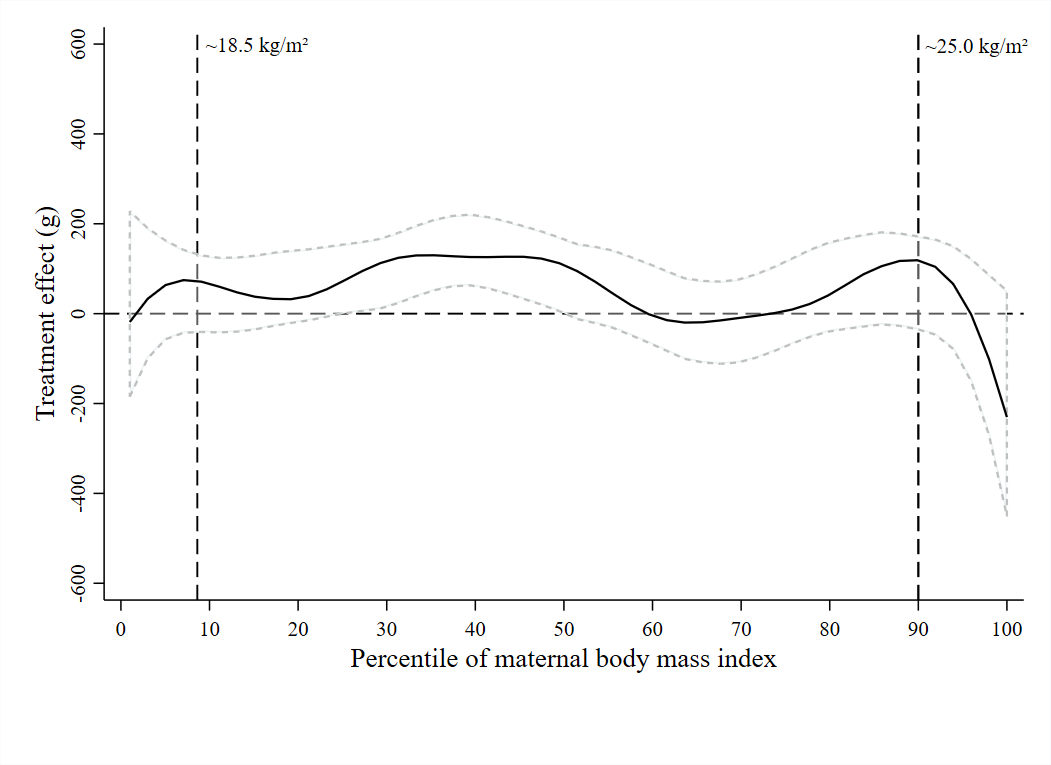

Supplement: S1 Fig — The estimated difference in birth weight between the women who received the BEP supplement and IFA (intervention) and those who received only iron and folic acid (control) is shown as a function of the percentiles of maternal BMI. The zero line indicates no efficacy of BEP. The positive y values indicate a higher birth weight in the intervention group, and the negative y values indicate a lower birth weight. The central solid black line represents the smoothed treatment efficacy, with upper and lower dashed 95% confidence bands, using complete cases. BEP, balanced energy–protein; BMI, body mass index; IFA, iron–folic acid. (TIF) [file pmed.1004002.s005.tif]

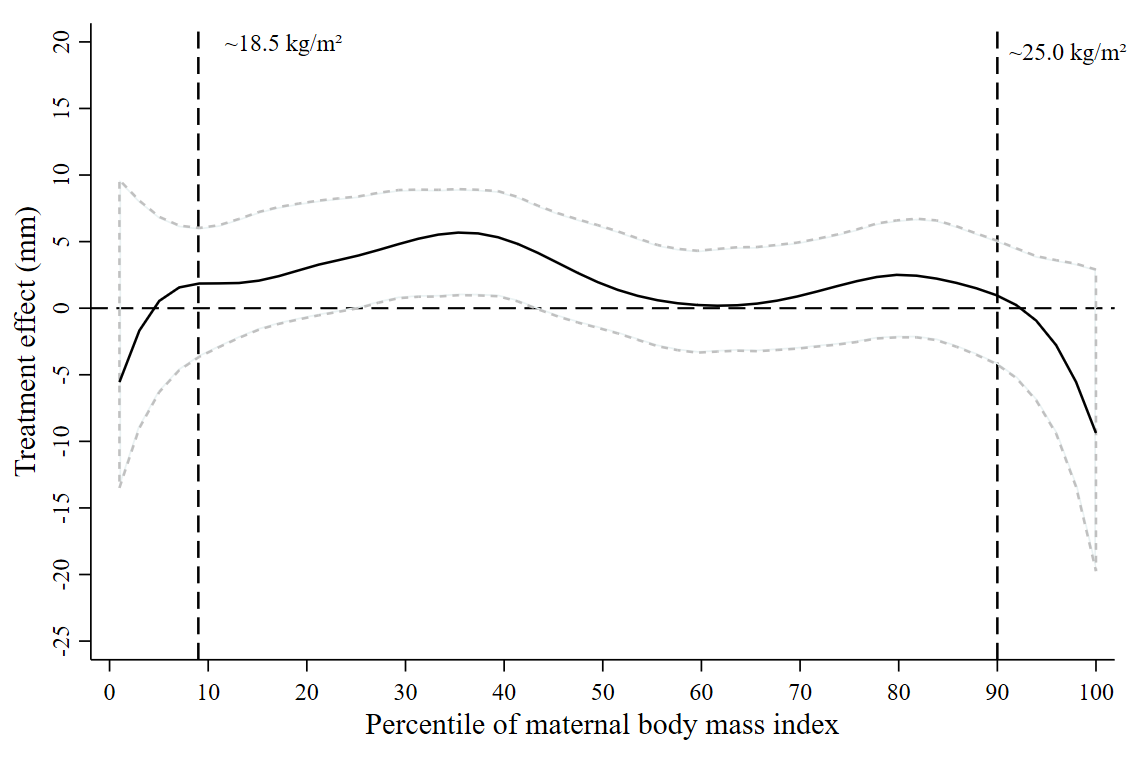

Supplement: S2 Fig — The estimated difference in birth length between the women who received the BEP supplement and IFA (intervention) and those who received only iron and folic acid (control) is shown as a function of the percentiles of maternal BMI. The zero line indicates no efficacy of BEP. The positive y values indicate a higher birth length in the intervention group, and the negative y values indicate a lower birth length. The central solid black line represents the smoothed treatment efficacy, with upper and lower dashed 95% confidence bands, using complete cases. BEP, balanced energy–protein; BMI, body mass index; IFA, iron–folic acid. (TIF) [file pmed.1004002.s006.tif]
