# Supplementary material for: Prenatal fortified balanced energy-protein supplementation and birth outcomes in rural Burkina Faso: A randomized controlled efficacy trial
Source: PLoS Med. 2022 May 13;19(5):e1004002. doi: 10.1371/journal.pmed.1004002 (PMC9140265; doi:10.1371/journal.pmed.1004002)
Supplement: Attachment — Submitted filename: dekok.pdf [file pmed.1004002.s007.pdf]

This paper describes a clinical trial of food supplementation and birth outcomes in Burkina Faso.

I was asked for a statistical report and I interpret that to include all aspects of the design and conduct of the study.

Points of detail

Page 8 Given the description of the randomisation scheme it is hard to see quite how the arms ended up with a difference of $960 - 937 = 23$ as page 14 suggests. This sort of thing can happen but the authors might give a few more details.

Page 8 Achieving complete masking to treatment arm is always difficult so I would not worry too much about it. I would suggest expanding on 'partially blinded'. What exactly does that imply for each of the study members listed?

Page 11 This refers to 'All SAEs (eg miscarriage and stillbirth). This implies that there was at least one other SAE type being monitored. However page 16, Table 4, only reports on foetal death and stillbirth and not on anything else. I would have classified this as a low–risk trial but SAEs can happen so I think we should see a report, perhaps in the supplement, of what SAEs were looked for and how many were found even if that means reporting lots of zeroes. I assume for instance that maternal death would have been an SAE and I assume none occurred but that is not explicitly stated in the article as far as I can see.

Page 15 and elsewhere I would avoid the $\pm$ notation as it can be ambiguous. More important the guidelines usually ask for absolute as well as relative frequencies. I am not convinced that with percentages to one decimal place the reader can unambiguously back–calculate them so I would include absolute numbers here and in other tables. This would be beneficial for subsequent meta–analysts who might want to use risk ratios or odds ratios in their analysis. Table 3 does have absolute numbers.

Page 19 Is there any explanation for the rather unusual increase from the 70 percentile onwards?

The fact that a woman took part whose disability meant her height could not be determined indicates that this study had a very inclusive attitude to participation.

Point of more substance

Looking at Table 4 the thing which strikes me as someone with little knowledge of neonatal anthropometry or obstetrics is that the various outcomes are all telling us the same thing and the authors' choice of SGA as their primary outcome just happens to be the one which failed to reach some arbitrary level of statistical significance. Increasing birth weight by about 1.6% and gestation by about 0.5% may be small but they have generated reductions in low birth weight and pre–term delivery. The authors are, of course, correct in sticking to their pre–specified choice of outcome but I wonder whether they are placing too little weight on the secondaries?

Summary

Mostly for clarification.

Michael Dewey
