# Supplementary material for: Prenatal fortified balanced energy-protein supplementation and birth outcomes in rural Burkina Faso: A randomized controlled efficacy trial
Source: PLoS Med. 2022 May 13;19(5):e1004002. doi: 10.1371/journal.pmed.1004002 (PMC9140265; doi:10.1371/journal.pmed.1004002)
Supplement: Attachment — Submitted filename: BEP and birth outcomes_Response to reviewers.docx [file pmed.1004002.s008.docx]

Beryne Odeny, PhD
Associate Editor, *PLOS Medicine*

April 4, 2022

RE: PMEDICINE-D-22-00628R1

Dear dr. Beryne Odeny,

Thank you for your email dated 23^rd^ March 2022 enclosing the editors’ and reviewers’ comments on our manuscript entitled ‘Prenatal fortified balanced energy-protein supplementation and birth outcomes in rural Burkina Faso: A randomised controlled efficacy trial’ (PMEDICINE-D-22-00628R1). The authors are grateful for the valuable feedback and the opportunity to revise the manuscript. We have carefully reviewed the comments and have incorporated most of the suggestions accordingly. Our reviewer responses are given by a point-by-point manner, including relevant page and line numbers of the “clean” manuscript text.

We hope the revised manuscript is now suitable for publication in *PLOS Medicine* and look forward to hearing from you in due course.

Yours sincerely,

Prof. dr. Carl Lachat

Corresponding author

**Requests from the editors:**

Thank you for your meticulous comments, which have greatly strengthened the readability of our manuscript.

1) The title needs revision. Please remove the country from the subtitle and place it right before the colon, i.e, “… in rural Burkina Faso: A randomized controlled efficacy trial”

**Response:** Thank you for the suggestion, we have changed the title (p1, lines 1-3).

2) Data availability statement requires revision - If the de-identified participant data are not freely available, please describe briefly the ethical, legal, or contractual restriction that prevents you from sharing it. Please also include an appropriate contact (web or email address) for inquiries (this cannot be a study author).

**Response:** The data availability statement has been revised upon request:

“The informed consent form does not allow sharing of personal data outside the research team. Request to access data need to be directed to the ethics committee of Ghent University Hospital through [ethisch.comite@uzgent.be](mailto:ethisch.comite@uzgent.be). Supporting study documents, including the study protocol and questionnaires, are publicly available on the study’s website: <https://misame3.ugent.be/>.” (p23-24, lines 452-455)

3) Abstract:
a) Please provide the follow-up time and dates of the study
b) Please provide the number of participants lost to follow up in each arm
c) Please include a summary of adverse events if these were assessed in the study.
d) Please remove “trial registration” after abstract conclusion and include it the abstract “methods and findings”

**Response:** Thank you for the suggestions to improve the abstract and include more detailed information.

a) The follow-up time and dates have been added (p3, lines 49-50).
b) The number of participants lost to follow up in each arm has been added (p3, lines 51-52).
c) A summary of adverse events has been added (p3, lines 60-61).
d) The trial registration has been moved and placed under the “Methods and findings” sub-section of the abstract (p13, lines 305-306).
4) Thank you for providing your CONSORT checklist. Please replace the page numbers with paragraph numbers per section (e.g. "Methods, paragraph 1"), since the page numbers of the final published paper may be different from the page numbers in the current manuscript.

**Response:** As requested, a new CONSORT checklist has been uploaded with the respective manuscript section and paragraph number.

5) Please label the CONSORT checklist as S1 Checklist.

**Response:** The CONSORT checklist is now labeled as “S1 Checklist”.

6) Please move the Ethical statement to the Methods section.

**Response:** The Ethical statement has been moved to the Methods section (p7, lines 147-148).

7) Please replace the term “compliance” with “adherence” where it is used to refer to treatment adherence.

**Response:** We have replaced “compliance” with “adherence” throughout the manuscript text.

8) Replace “fetus” rather than “foetus”

**Response:** We have replaced “foetus” with “fetus” throughout the manuscript text and Figure 1.

9) Figure 1 should be labeled “Fig 1. CONSORT flowchart” or similar

**Response:** The label of Figure 1 has been changed to “Fig 1. CONSORT flowchart”.

10) In the tables, please use “p value” instead of “P”

**Response:** We have replaced “P” with “p value” in all tables.

11) In the tables, please define symbol ∆

**Response:** The symbol ∆ has been defined as “Unadjusted and adjusted group differences (∆)” in the relevant tables.

12) Please remove the ‘Funding” and “Conflict of interest”, from the end of the main text. In the event of publication, this information will be published as metadata based on your responses to the submission form

**Response:** As requested, the “Funding” and “Conflict of interest” have been removed from the main text.

13) References
a) Please ensure journal name abbreviations consistently match those found in the National Center for Biotechnology Information (NCBI) databases
b) Please provide access dates for references with web links.
c) Ref #37 is incomplete – please include the journal name

**Response:**

a) The journal name abbreviations have been adjusted according to the NCBI databases.
b) The access dates for references with web links have been added (references 8, 19, 28)
c) Ref #37 has been updated, which is now reference #38.

**Comments from the reviewers:**

**Reviewer #1:** This manuscript is an extremely well-conducted study of maternal supplemental in a vulnerable population in the Sahel. The writing and documentation are coherent, thorough and transparent.

The daily observed administration of the supplement leaves no doubt that the findings genuinely reflect what occurs when the supplement is consumed.

My major ask is reconsider reporting other important outcomes in another manuscript.  Make this a complete, coherent story about all of the effects of BEP supplementation in pregnancy.  Tell us what BEP does to maternal weight gain and quality of life, birth outcomes and postnatal outcomes in a single story. Allow readers to quickly understand the full impact of the BEP supplement.

**Response:** Thank you for your constructive and insightful comments. We are pleased to receive your positive feedback regarding the public health value, validity, and documentation of the prenatal phase of the MISAME-III efficacy trial.

To respond to your first suggestion, we propose to keep the focus on the birth outcomes in this manuscript. First, including all outcomes of the MISAME-III trial (i.e. including those related to maternal health and post-natal growth) will require an additional discussion on different biological mechanisms related to maternal and child health. This would also involve a different statistical analysis (i.e. baseline prognostic factors and four, rather than two (prenatal) study arms due to the factorial design). Adding these considerations, with adequate detail, will in our opinion not do justice to the clarity of the manuscript. Second, at the time of writing, data collection on the postnatal phase of the MISAME-III was just completed. The present manuscript aimed to disseminate findings on birth outcomes timely, to provide additional guidance to the ongoing efforts of the WHO regarding vulnerable newborns.

Minor additions to strengthen the manuscript would be to include a description of the context of the participants with regard to common infections. Is this area malaria endemic? What have other studies found with regard to sexually transmitted diseases? How much HIV is seen in the study context? Are there other endemic infectious conditions?

**Response:** Thank you for the suggestion to include a description of the context with regard to common infections. We have contacted the regional health office of Houndé district and have added the statistics from the six health care centres in 2021 to provide additional context (p7-8, lines 159-162, reference 19).

Was there any seasonal effect seen? Is there a season with more food insecurity and did the BEP supplement given more benefits during these times?

**Response:** The climate in the study setting is Sudano-Sahelian, with the dry season conventionally running from September-October to April (p7, line 158-159). Food shortage is mostly experienced between June-September, which is the period between the depletion of the cereal stocks and the next harvest.

We have tested an interaction term between the intervention and the season of delivery (lean season: June-September) and did not find a significant difference (p value = 0.50) on the primary outcome SGA. The results are presented in Table S3. In addition, to answer your question in this rebuttal letter, we have repeated this analysis for all the secondary outcomes showing similar results (p values > 0.10). To summarize, we found no evidence that supports an increased benefit of the BEP supplement during the period with higher risk of food insecurity.

**Reviewer #2:** Michael Dewey

This paper describes a clinical trial of food supplementation and birth outcomes in Burkina Faso.

I was asked for a statistical report and I interpret that to include all aspects of the design and conduct of the study.

**Response:** Thank you for your detailed review of the methodological and statistical aspects of our efficacy trial. We highly appreciate your critical standpoint, comments, and suggestions to improve the manuscript text.

**Points of detail**

**Page 8** Given the description of the randomisation scheme it is hard to see quite how the arms ended up with a difference of 960 – 937 = 23 as page 14 suggests. This sort of thing can happen but the authors might give a few more details.

**Response:** You have raised an important remark. In theory, our block randomization scheme would have led to similar sample sizes in the control and intervention arms. However, due to the pragmatic nature of the study inclusion and subsequent randomization, we ended up with a slight difference between the study groups. To clarify, at the first ANC visit, certain women were confirmed as pregnant by a urinary pregnancy test and randomized to prenatal control and intervention arms (i.e., “Enrollment” in Figure 1), but their pregnancy was not deemed vital during the subsequent ultrasound examination (due to e.g. molar pregnancy, miscarriage). Although these woman received a randomization code, the woman did not receive any supplements as they did not meet the *a priori* study inclusion criteria. They were therefore not included at the “Allocation” stage in Figure 1, which represents only the number of women who actually received either BEP + IFA or IFA. We have added this detail for clarification in the manuscript (p13, lines 312-313).

**Page 8** Achieving complete masking to treatment arm is always difficult so I would not worry too much about it. I would suggest expanding on ‘partially blinded’. What exactly does that imply for each of the study members listed?

**Response:** We agree that this terminology ‘partially blinded’ might be confusing. We decided to drop this term and change the text according to the updated WHO bias assessment classification: “Outcome assessors (study physician, midwives, and field supervisors) were different from study collaborators (trained village-based project workers) who distributed the study supplements. However, given the non-blinded nature of the study, outcome assessors could have been aware of the study group allocation by asking the mother.” (p8, lines 175-178).

**Page 11** This refers to ‘All SAEs (eg miscarriage and stillbirth)’. This implies that there was at least one other SAE type being monitored. However page 16, Table 4, only reports on foetal death and stillbirth and not on anything else. I would have classified this as a low-risk trial but SAEs can happen so I think we should see a report, perhaps in the supplement, of what SAEs were looked for and how many were found even if that means reporting lots of zeroes. I assume for instance that maternal death would have been an SAE and I assume none occurred but that is not explicitly stated in the article as far as I can see.

**Response:** Thank you for your request to clarify the screening for SAEs in our study. We indeed screened for fetal loss, stillbirth, and maternal death. The later did not occur in our study. We have added this detail in the text (p11, lines 252-253; p16, line 323).

**Page 15** **and elsewhere** I would avoid the ± notation as it can be ambiguous. More important the guidelines usually ask for absolute as well as relative frequencies. I am not convinced that with percentages to one decimal place the reader can unambiguously back-calculate them so I

would include absolute numbers here and in other tables. This would be beneficial for subsequent meta-analysts who might want to use risk ratios or odds ratios in their analysis. Table 3 does have absolute numbers.

**Response:** Thank you for this valuable suggestion, we have added the absolute numbers in all tables (including supplementary tables) to provide more precise information. The “±” sign for SD is explained in the footnote and we checked other recent articles published in *PLOS Medicine*, which use the same notation.

**Page 19** Is there any explanation for the rather unusual increase from the 70 percentile onwards?

**Response:** Figure S2 is an exploratory analysis, which indicates that at both tails of the birth length distribution, the trial efficacy was greatest. Nevertheless, the overall treatment effect on birth length was modest, and the biological mechanism for the increase from the 70^th^ percentile is unfortunately not clear.

**Point of more substance**

Looking at Table 4 the thing which strikes me as someone with little knowledge of neonatal anthropometry or obstetrics is that the various outcomes are all telling us the same thing and the authors' choice of SGA as their primary outcome just happens to be the one which failed to reach some arbitrary level of statistical significance. Increasing birth weight by about 1.6% and gestation by about 0.5% may be small but they have generated reductions in low birth weight and pre-term delivery. The authors are, of course, correct in sticking to their pre-specified choice of outcome but I wonder whether they are placing too little weight on the secondaries?

**Response:** Indeed, we chose to stick to our pre-specified Statistical Analysis Plan with the *a priori* defined primary and secondary outcomes to avoid susceptibility to selectively report child anthropometric birth outcomes. The primary outcome of small-for-gestational age (SGA) was chosen as the clinical shift from (low) birth weight to small-for-gestational age is nowadays scientifically well established (i.e., former is highly correlated with gestational duration). Our findings suggest that BEP supplementation leads to a slightly longer gestation and, as a result, to slightly heavier and longer newborns. We deliberately prefer not to emphasize the effects on these secondary outcomes because i) their exploratory nature; 2) the small effect sizes.

Nevertheless, we report on both the primary and secondary outcomes in the abstract, results, discussion, and summary of the manuscript and thereby, with our findings, hope to transparently contribute to evidence-based decision making.

**Reviewer #3:** This is an important study on a public health intervention, where despite global guidance, there is paucity of information from well-designed population based studies. This is one of the well-controlled and designed studies with early enough introduction the BEP, assessment of compliance and robust statistical analysis. The field methods had strong quality assurance methodology and the results therefore provide strong evidence that adds to our understanding of maternal nutrition interventions.

The paper could be strengthened by attention to a few issues in the description of the design and analysis part of the study

**Response:** Thank you for your positive review of the design and analysis of the MISAME-III trial. We highly appreciate your critical insights and critical remarks, which have increased the clarity of our manuscript.

1. Why was a 7% point reduction in SGA births chosen for sample size and power estimation? A lower figure could well have yielded a larger sample size and relevant impact

**Response:** On the basis of an estimated 32% prevalence of SGA in the previous MISAME-II trial, which was based on a combination of ultrasound and last menstrual period measurements, and a treatment effect of ̴ 4 percentage points (not significant) in SGA prevalence, we estimated that a 7 percentage point SGA reduction would be a realistic and clinically relevant difference. We refer to the Cochrane review by Ota and colleagues (2015) who found an impact of BEP supplementation on SGA of RR=0.79. Applying this RR to a baseline prevalence of 32% leads to an anticipated SGA prevalence in the intervention group of 25%, which corresponds to a risk difference of 7 percentage points. We preferred to express the minimally detectably difference as a risk difference to be consisted with the analytical model.

In fact, in the current study, the baseline prevalence of SGA was estimated to be 28% in the control group, which was more accurately assessed by ultrasound only in the MISAME-III trial. Thus, recalculating the sample size using an SGA prevalence of 28% and a 7 percentage point reduction (*α* = 0.05, *β* = 0.80) would have resulted in a required sample of 592 per study arm (*n* = 1184 in total). Hence, our original sample size calculation provides sufficient power to detect the *a priori* defined difference in SGA.

2. The authors focused on SGA as the primary outcome and found significant impact on preterm births. Given the excess risk of mortality and other health and growth outcomes in early infancy among newborn infants with are both preterm and SGA, why wasn't the composite outcome assessed as an outcome, even if secondary?

**Response:** Thank you for this interesting questions. These composite outcomes were not pre-registered in our Statistical Analysis Plan; hence, we have not reported on them to avoid cherry-picking of study outcomes and perform underpowered analysis. The present study’s outcomes were defined in an expert consultation held at the Bill & Melinda Gates Foundation (2017), and along the way, we were unable to change these. As we were also interested to see the effect on infants at increased risk, we had a look at the WHO ‘vulnerable newborns’ categories in our study, which appeared to represent very small sample sizes:

- Extremely vulnerable (preterm, LBW and SGA): 1% control, 1% intervention;
- Severely vulnerable (preterm, LBW, not SGA): 3% control, 1% intervention;
- Highly vulnerable (preterm, not LBW, not SGA): 1% control, 1% intervention.

Therefore, we decided not to report on these in the manuscript.

3. The authors state that there was no displacement of food by the BEP supplement but do not present any intake data. If there are dietary intake data, these should be presented.

**Response:** Thank you for your suggestion. This data is indeed important to put the trial’s prenatal results into perspective. We conducted a cross-sectional study and collected a single multiple-pass 24h recall from 470 pregnant women from the MISAME-III study, and the results showed that there was no displacement of food by the BEP supplement. The findings of this sub-study were previously published and we have provided the reference in the manuscript so readers can refer to this article for more details (p21 lines 402-403; reference 43).
